# Wait-and-see treatment strategies for rectal cancer patients with clinical complete response after neoadjuvant chemoradiotherapy: a systematic review and meta-analysis

**DOI:** 10.18632/oncotarget.8622

**Published:** 2016-04-06

**Authors:** Jun Li, Lunjin Li, Lin Yang, Jiatian Yuan, Bo Lv, Yanan Yao, Shasha Xing

**Affiliations:** ^1^ General Surgery Department and Central Laboratory, Affiliated Hospital/Clinical Medical College of Chengdu University, Chengdu, People's Republic of China; ^2^ Pharmacy Department, Affiliated Hospital/Clinical Medical College of Chengdu University, Chengdu, People's Republic of China; ^3^ Department of Pathology, Cancer Hospital, Chinese Academy of Medical Sciences, Beijing, People's Republic of China; ^4^ Department of General Surgery, The First Affiliated Hospital of Sun Yat-sen University, Guangzhou, People's Republic of China; ^5^ Central Laboratory, Affiliated Hospital/Clinical Medical College of Chengdu University, Chengdu, People's Republic of China

**Keywords:** rectal cancer, clinical complete response, neoadjuvant chemoradiotherapy, wait-and-see

## Abstract

Wait-and-see treatment strategies may benefit rectal cancer patients who achieve a clinical complete response (cCR) after neoadjuvant chemoradiotherapy (NCRT). In this study, we analyzed data from 9 eligible trials to compare the oncologic outcomes of 251 rectal cancer patients achieving a cCR through nonsurgical management approaches with the outcomes of 344 patients achieving a pathologic complete response (pCR) through radical surgery. The two patient groups did not differ in distant metastasis rates or disease-free and overall survival, but the nonsurgical group had a higher risk of 1, 2, 3, and 5-year local recurrence. Hence, we concluded that for rectal cancer patients achieving a cCR after NCRT, a wait-and-see strategy with strict selection criteria, an appropriate follow-up schedule, and salvage treatments achieved outcomes at least as good as radical surgery. Long-term randomized and controlled trials with more uniform inclusion criteria and standardized follow-up schedules will help clarify the risks and benefits of wait-and-see treatment strategies for these patients.

## INTRODUCTION

The standard treatment for locally advanced rectal cancer is neoadjuvant chemoradiotherapy (NCRT) followed by radical surgery (total mesorectal excision, TME) 4-8 weeks later [1]. Several studies have demonstrated superior local control with this strategy, which even leads to a clinical complete response (cCR), defined as the absence of detectable residual tumor cells, in a substantial proportion of patients treated by NCRT. Nevertheless, a wait-and-see policy might be more beneficial for rectal cancer patients with no residual tumor or involved lymph nodes after NCRT [2, 3]. The first study of the wait-and-see policy, which entails observational management of rectal cancer patients with a cCR after NCRT, was reported by Habr-Gama et al. [4]. A series of retrospective studies from the same group [3] showed that patients with a cCR who were managed with an observational approach had survival rates similar to patients with a pathologic clinical response (pCR) who underwent radical surgery. Although this was a small study, the wait-and-see policy attracted much interest among clinicians, and additional studies [2, 5–11] have confirmed the efficacy of an observational approach using MRI and endoscopy with biopsy to evaluate clinical responses.

Patients treated using the wait-and-see policy who achieve a complete tumor response avoid the risk of surgical morbidity and mortality. However, guidelines regarding the use of cCRs to predict pCR and develop a clinical, pathologic, and imaging follow-up schedule are lacking. For this reason, despite having cCR, patients who did not undergo an operation face a high risk of local recurrence (LR), even though a substantial proportion of patients suffering LR can be treated through salvage treatments. Additionally, the long-term efficacy of this wait-and-see approach is unidentified clearly, which limits its use.

Here, we conducted a systematic review and meta-analysis of the medical literature related to nonoperation management of rectal cancer after NCRT to determine oncologic outcomes of the wait-and-see strategy.

## RESULTS

**Figure 1:**
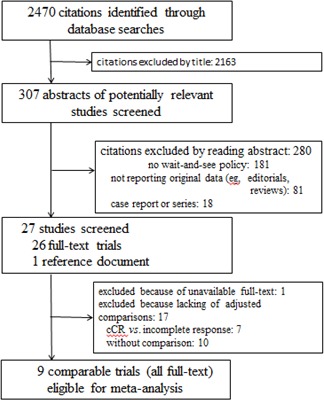
Study selection process for systematic review and meta-analysis

**Figure 2:**
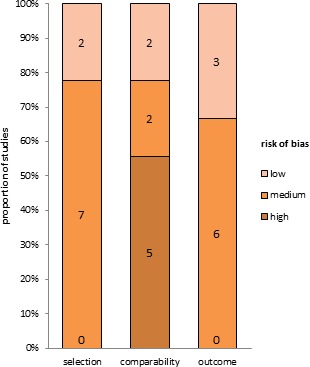
Quality assessment using the Newcastle-Ottawa Scale for risk of bias of studies included in the meta-analysis The absolute numbers of studies are shown in boxes. Low risk of bias is indicated by four stars for selection, two stars for comparability, and three stars for outcome. Medium risk of bias is indicated by two or three stars for selection, one for comparability, and two for outcome. High risk of bias is indicated by one star for selection or outcome, or zero for any of the three components. Studies were eligible for meta-analysis if LR and distant metastasis data were included. In selection of patients, no articles were high risk, 7 were medium risk, and 2 were low risk. The risk of bias in outcome was similar to that for patient selection (0, 6, and 3, respectively). For comparability, there were 5 high risk, 2 medium risk, and 2 low risk articles. The funnel pots used to assess publication bias indicated no obvious bias.

Our initial search identified 2, 470 citations (Figure 1). 2, 163 citations with titles that did not satisfy eligibility criteria were excluded. After reading the abstracts of the remaining articles, 26 full-text trials were read (Table 1). Information was also used from one presentation abstract for which full text was not available [29]. Several papers by Habr-Gama and colleagues describing studies of Brazilian patients were examined [4, 14, 15, 18, 20, 22, 23, 26], but only one of them that included all data of interest was recruited for this meta-analysis [4]. Finally, nine comparative studies of 26 trials which focused on oncologic outcome in patients with cCR in a wait-and-see group compared to those with pCR in a radical surgery group were identified [2, 4–9, 11, 13] (Table 2). Tables 2 and 3 show the main characteristics of these nine comparative studies. Figure 2 shows the risk of bias.

**Table 1:** Clinical characteristics and oncologic outcome of all recent studies focused on wait-and-see policy

Study (year)	No. of OB (inclusion interval)	Age (range)	Gender (M/F)	Distance from AV(cm)	cT stage	cN stage	Dose of Radiation (No. of Patients)	Chemo	Time of Assessment (weeks)	Follow-up (months, range)	LR			Salvage Treatment	1-Year Survival(%)		2-Year Survival(%)		3-Year Survival(%)		4-Year Survival(%)		5-Year Survival(%)	
											No.	%	Time (months)		DFS	OS	DFS	OS	DFS	OS	DFS	OS	DFS	OS
Rupinski et al [12] (2015)	11 (2012-2015)	NS	NS	NS	NS	NS	50Gy(7); 25Gy(4); 25Gy + 4Gy boost(1)	5-FU+LV	8-12	median 7 (NS)	NS	NS	NS	NS	NS	NS	NS	NS	NS	NS	NS	NS	NS	NS
Araujo et al [9](2015)	42 (2002-2013)	median 63.6 (NS)	17/25	NS	NS	NS	45.0-50.4Gy	5-FU+LV; capecitabine	6-8	median 47.7 (NS)	8	19	median 24.5 (8-32)	3 APR (1 R1); 1 LAR; 1 refused	40(95.2)	NS	36(85.7)	NS	31(73.8)	NS	30(71.4)	NS	60.9	NS
Li et al [2](2015)	30 (2006-2013)	median 62 (55-82)	18/12	mean 3.5 (0-7)	T1-4	N0/N+	50Gy ; 25Gy	capecitabine	8-10	mean 58 (19-108)	2	6.7	median 22 (18-26)	1 TME; 1 LE	30(100.0)	30(100.0)	29(96.7)	30(100.0)	28(93.3)	30(100.0)	28(93.3)	30(100.0)	27(90.0)	30(100.0)
Lee et al [13](2015)	8 (2006-2011)	? 70 (50-82)	7/1	? 4(2-4)	T2-4	N0/N+	50.4Gy	5-FU-based	6-10	NS	2	25	NS	1 palliative Chemo; 1 APR	6(75.0)	NS	6(75.0)	NS	6(75.0)	NS	NS	NS	NS	NS
Appelt et al [10](2015)	40 (2009-2013)	NS	NS	NS	T2-3	N0/N+	60Gy to rectal wall + 50Gy to nodes	tegafur-uracil(UFT)	6	median 23.9 ( 15.3-31.0)	9	22.5	median 10.4 (8.0-13.6)	9 RS	34(85.0)	40(100.0)	31(77.5)	40(100.0)	—	—	—	—	—	—
Smith RK et al [8](2015)	18 (2001-2013)	mean 62.3 (NS)	15/3	mean 4.1	T1-3	N0/N+	NS	5-FU; capecitabine	7-24	mean 68.4 (NS)	1	5.6	9.4	1 radiation	17(94.4)	18(100.0)	17(94.4)	18(100.0)	16(88.9)	18(100.0)	16(88.9)	18(100.0)	16(88.9)	18(100.0)
Habr-Gama et al [14](2014)	90 (1991-2011)	mean 58.9±57.5 (NS)	60/30	mean 3.3±2.0	T2-4	N0/N+	50.4-54Gy; 45Gy + 9Gy boost	5-FU+LV	8	median 60 (12-233)	28	31	median 9.5 (3-64)	25 RS; 1 BT; 1 (LR and DM) Chemo; 1 no therapy	73(81.1)	NS	67(74.4)	NS	67(74.4)	NS	64(71.1)	NS	63(70.0)	NS
Habr-Gama et al [15](2013)	47 (2006-2010)	mean 60.2±12.7 (NS)	27/20	mean 3.7±1.7	T2-4	N0/N+	45Gy + 9Gy boost	5-FU+LV	10	median 56 (NS)	12	25.5	median 45 (16-144)	early LR: 3 RS; 3 FTLE; 1 BT + APR; 1 Chemo. late LR: 2 RS; 2 FTLE	35(74.5)	NS	NS	NS	NS	NS	NS	NS	NS	NS
Seshadri et al [11](2013)	23 (1991-2008)	median 50 (25-71)	14/9	median 3 (0-6)	T2-3	NS	NS	Ns	4-6	median 72 (12-180)	7	30.4	median 12 (5-30)	3APR; 2 refused; 1 CAA; 1 LAR;	17(73.9)	23(100.0)	16(69.6)	21(91.3)	15(65.2)	23(100.0)	14(60.9)	17(73.9)	14(60.9)	17(73.9)
Smith JD et al [7](2012)	32 (2006-2010)	median 70 (NS)	18/14	? 6 (0.5-12)	T2-3	N0/N+	median 50.4Gy (45.0-56.0)	5-FU; capecitabine	4-10	median 28 (9-70)	6	18.8	median 11.5 (7-14)	2 APR; 3 LAR;1 TAE then APR (R1)	28(87.5)	32(100.0)	28(87.5)	31(96.9)	—	—	—	—	—	—
Perez et al [16](2012)	16 (2005-2009)	NS	Ns	Ns	Ns	Ns	45 Gy + 9Gy boost	5-FU+LV	12	mean 42.6±15.6 (NS)	1	6.3	11	1 TSLE	15(93.8)	16(100.0)	14(87.5)	15(93.8)	14(87.5)	15(93.8)	—	—	—	—
Dalton et al [6](2012)	6 (2004-2009)	median 64 (54-71)	5/1	mean 5.06±3.27	T2-4	N0/N+	45Gy	capecitabine	6-8	mean 25.5 (12-45)	0	0	No LR	—	6(100.0)	6(100.0)	6(100.0)	6(100.0)	—	—	—	—	—	—
Maas et al [5](2011)	21 (2004-2010)	median 65 (49-79)	14/7	mean 2.9 (0-10)	T1-4	N0/N+	50.4Gy	capecitabine	6-8	median 15 (5-67)	1	4.8	22	1 TEM	21(100.0)	21(100.0)	19(90.5)	21(100.0)	—	—	—	—	—	—
Lambregts et al [17](2011)	19 (NS)	NS	NS	NS	T1-4	N0/N+	50.4Gy	capecitabine	6-8	median 22 (12-60)	1	5.3	22	1 TEM	19(100.0)	19(100.0)	18(94.7)	NS	NS	NS	NS	NS	NS	NS
Habr-Gama et al [18](2011)	67 (1991-2009)	NS	NS	NS	T2-4	N0/N+	50.4-54Gy; 45Gy + 9Gy boost	5-FU-based	8	mean 65 (NS)	8	11.9	median 39 (NS)	3 TSLE; 1 BT; 4 RS	NS	NS	NS	NS	NS	NS	NS	NS	48(72.0)	64(96.0)
Hughes et al [19](2010)	10 (1993-2005)	median 78.5(NS)	NS	NS	T3-4	NS	45Gy	5-FU+LV; 5-FU; capecitabine; irinotecan; oxaliplatin	6-8	NS	6	60.0	NS	NS	NS	NS	NS	NS	NS	NS	NS	NS	NS	NS
Habr-Gama et al [20](2009)	22 (2005-2008)	mean 57.6±11.8(NS)	14/5	mean 4.22±1.25	T2-3	N0/N+	45.0Gy + 9Gy boost	5-FU+LV	10	mean 23.2±10.7 (NS)	22	13.6	NS	3 RS	19(86.4)	22(100.0)	NS	NS	NS	NS	NS	NS	NS	NS
Lim et al [21](2007)	27 (1998-2005)	median 76(49-94)	35/13	NS	NS	NS	52Gy (25-61.4Gy)	5-FU+LV; 5-FU	4-6	49 (NS)	9	39.0	NS	NS	NS	NS	NS	NS	NS	NS	NS	NS	NS	NS
Habr-Gama et al [22](2006)	99 (1991-2005)	mean 60.8±14.1 (NS)	47/52	mean 3.9±1.7	T2-4	N0/N+	50.4Gy	5-FU+LV	8	mean 59.7±45.7 (NS)	6	6.1	median 49.5 (18-79)	2 APR; 1 LAR; 1 LE; 1 BT; 1 APR then Chemo (LR and DM)	98(99.0)	99(100.0)	91(91.9)	94(94.9)	91(91.9)	94(94.9)	89(89.9)	94(94.9)	87(87.9)	93(93.9)
Habr-Gama et al [23](2006)	99 (1991-2005)	NS	NS	NS	NS	NS	50.4Gy	5-FU+LV	8	NS	6	6.1	mean 96 (NS)	5 salvage surgery (NS); 1 unclear (LR and DM)	NS	NS	NS	NS	NS	NS	NS	NS	NS	NS
Wang et al [24](2005)	80 (1978-1997)	NS	NS	NS	NS	NS	mostly 52Gy (40-60Gy)	NO	median 4 months (1-11, from RT start)	NS	62	77.5	median 18 (3-108)	Ns	34.0% (LR)	NS	59.0% (LR)	NS	NS	NS	NS	NS	79.0% (LR )	NS
Habr-Gama et al [4](2004)	71 (1991-2008)	mean 58.1 (35-92)	36/35	mean 3.6 (0-7)	T2-4	N0/N+	50.4Gy	5-FU+LV	8	mean 57.3 (12-156)	2	2.8	median 60 (56-64)	1 TEM; 1 BT	71(100.0)	71(100.0)	70(98.6)	71(100.0)	70(98.6)	71(100.0)	69(97.2)	71(100.0)	68(95.8)	71(100.0)
Nakagawa et al [25](2002)	10 (1993-1997)	median 50.5 (23-70)	NS	NS	NS	NS	45Gy (1); 50.4Gy (9)	5-FU+LV	3-4	NS	8	80.0	meidan 6 (3.7-8.8)	6 RS; 1 no surgery (LR and DM); 1 refused	2(20.0)	NS	NS	NS	NS	NS	NS	NS	NS	NS
Habr-Gama et al [26](1998)	30 (1991-1996)	NS	NS	NS	NS	NS	50.4Gy	5-FU+LV	6-8	NS	NS	NS	NS	NS	NS	NS	NS	NS	NS	NS	NS	NS	NS	NS
Rossi et al [27](1998)	6 (1993-1996)	NS	NS	NS	NS	NS	50.4Gy + boost (20-30Gy, 5) ; 45Gy(1)	5-FU + LV + levamisole	4	median 23 (8-40)	5	83.3	median 8 (1-8)	4 APR; 1 vaginal resection	1(16.7)	6(100.0)	1(16.7)	5(83.3)	—	—	—	—	—	—
Gerard et al [28](1996)	28 (1986-1992)	NS	NS	NS	NS	NS	median 70Gy	NO	NS	median 46 (9-95) (from radiation)	7	25.0	median 18 (7-48)	1 APR; 2 laser; 4 unclear (LR and DM)	NS	28(100.0)	23(82.1)	27(96.4)	NS	NS	NS	NS	NS	NS

**Abbreviation:** ? =authors described unclearly. AV= anal verge. N+= positive clinical nodes status. NS=not stated. LR=local recurrence. DM=distant metastasis. DFS=disease free survival. OS=overall survival. APR= abdominoperineal resection. LAR=low anterior resection. CAA=colo-anal anastomosis. BT= brachytherapy. Chemo=chemotherapy. LE=local excision. TSLE=transanal local excision. TEM=transanal endoscopy microsurgery.

**Table 2:** Characteristics of included comparative studies

Studies (year)	No.of Patients		Age		Gender(M/F)		Clinical stage of Population		Distance From AV(range)		Type of NT	Time(weeks)		Type of Study
	OB	Surgery	OB	Surgery	OB	Surgery	OB	Surgery	OB	Surgery		Assessment of cCR	to Sugery	
Araujo et al [9](2015)	42	69	median 63.6 (NS)	median 60.1 (NS)	17/25	34/35	NS	NS	≤5cm(35); ≥5cm(7)	≤5cm(41); ≥5cm(28)	5-FU or CAPE+RT	6-8	6-8	prospective cohort study, single center
Li et al [2](2015)	30	92	median 62 (55-82)	median 56 (34-73)	18/12	60/32	TNM1-3	TNM1-3	mean 3.5 (0-7)	mean 3.8 (0-7)	CAPE+RT	8-10	8-10	prospective cohort study, multi-center
Lee et al [13](2015)	8	28	? 70(50-82)	? 64 (46-80)	7/1	21/7	NS	NS	? 4(2-4)	median 4 (0-8)	5-FU+RT	6-10	6-10	prospective cohort study, single center
Smith RK [8]et al (2015)	18	30	mean 62.3 (NS)	mean 60.4 (NS)	15/3	20/10	TNM1-4	TNM1-3	mean 4.1 (Ns)	mean 6.0 (Ns)	5-FU or CAPE+RT	7-24	7-24	retrospective cohort study, single center
Seshadri et al [11](2013)	23	10	mean 50(25-71)	mean 55 (30-69)	14/9	6/4	NS	NS	median 3 (0-6)	median 4 (0-7)	5-FU+RT	4-6	median 14 (5-44)	retrospective cohort study, single center
Smith JD et al [7](2012)	32	57	median 70 (NS)	median 60 (NS)	18/14	27/30	TNM1-3	TNM1-3	6 (0.5-12)?	? 7 (2-12)	5-FU+RT	4-10	median 6.9 (5-17)	prospective cohort study, single center
Dalton et al [6](2012)	6	6	median 64(54-71)	NS	5/1	Ns	NS	NS	mean 5.06±3.27	NS	NS	6-8	6-8	prospective cohort study, single center
Maas et al [5](2011)	21	20	median 65(49-79)	median 66 (37-81)	14/7	16/4	NS	NS	mean 2.9 (0-10)	mean 3.4 (0-9)	CAPE+RT	6-8	6-8	prospective cohort study, single center
Habr-Gama et al [4](2004)	71	22	mean 58.1(35-92)	mean 53.6 (25-73)	36/35	12/10	NS	NS	mean 3.6 (0-7)	mean 3.8 (2-7)	5-FU+RT	8	8	prospective cohort study, single center

**Abbreviations:** OB=observation. ? =authors described unclearly. AV= anal verge. NS= not stated. CAPE=capecitabine.

**Table 3:** Clinical stage before neoadjuvant therapy and LR, DM, and total failure rates in included studies

Studies (year)	NO.of Patients		OB Group						Surgery Group						Interval of Follow-Up (range)		No.of LR (%)		No.of DM (%)		No.of All Failure (%)	
	OB	Surgery	cT1	cT2	cT3	cT4	cN0	cN+	cT1	cT2	cT3	cT4	cN0	cN+	OB	Surgery	OB	Surgery	OB	Surgery	OB	Surgery
Araujo et al [9](2015)	42	69	NS	NS	NS	NS	NS	NS	NS	NS	NS	NS	NS	NS	median 47.7 (NS)	median 46.7 (NS)	8(19.0)	1(1.4)	7(16.6)	7(10.1)	12(28.5)	8(11.5)
Li et al [2](2015)	30	92	3	5	15	7	14	16	10	14	48	20	39	53	mean 58 (19-108)	mean 58 (18-108)	2(6.7)	2(2.2)	1(3.3)	6(6.5)	3(10.0)	8(8.7)
Lee et al [13](2015)	8	28	0	5	2	1	5	3	0	6	21	1	13	15	median 41 (6-80)	median 41 (6-80)	2(25.0)	1(3.6)	0	3(10.7)	2(25.0)	4(14.3)
Smith RK et al [8](2015)	18	30	1	1	16	0	11	7	0	4	25	1	18	12	mean 68.4 (NS)	mean 46.3 (NS)	1(5.6)	0	1(5.6)	1(3.3)	2(11.1)	1(3.3)
Seshadri et al [11](2013)	23	10	0	9	14	0	NS	NS	0	4	6	0	NS	NS	median 72 (12-180)	median 37 (12-180)	7(30.4)	0	3(13.0)	2(20.0)	10(43.5)	2(20.0)
Smith JD et al [7](2012)	32	57	0	10	22	0	14	18	0	11	39	0	20	31	median 28 (9-70)	median 42 (1-70)	6(18.8)	0	3(9.4)	3(5.3)	6(18.8)	3(5.3)
Dalton et al [6](2012)	6	6	0	1	4	1	1	5	NS	NS	NS	NS	NS	NS	mean 25.5 (12-45)	mean 39.3 (15-57)	0	0	0	0	0	0
Maas et al [5](2011)	21	20	1	5	13	2	6	15	0	1	17	2	3	17	median 15 (5-67)	median 35 (1-77)	1(4.8)	0	0	1(5.0)	1(4.8)	1(5.0)
Habr-Gama et al [4](2004)	71	22	0	14	49	8	55	16	0	1	19	2	16	6	mean 57.3 (12-156)	mean 48 (12-83)	2(2.8)	0	3(4.2)	3(13.6)	5(7.0)	3(13.6)

Of the 585 patients included in the nine comparative articles, 42.9% (251/585) belonged to the cCR with observation group and 57.1% (334) to the pCR with radical surgery group. The male/female ratio was 144/107 and 196/132 in the two groups, respectively. Observation group patients seemed to be older than those in the radical surgery group [2, 4, 7–9, 13]. Except for one observation group patient with liver metastasis in Smith et al. [8], no patients had distant metastasis (DM) according to the study descriptions. Most patients had medial/distal and locally advanced rectal cancer. Patients received doses of radiation ranging from 45 to 54Gy. Chemotherapy regimens were based on 5-FU with/without capecitabine and LV, or capecitabine alone. The interval between NCRT completion and assessment/surgery was usually 6-8 weeks.

16.3% (41/251) of observation group patients suffered a treatment failure (LR and/or DM); 11.5% (29) of these had LR and 7.2% (18) had DM. 79.3% (23/29) patients received salvage treatments. These 29 patients with LR were treated as follows: 34.5% (14/29) with LR were treated with radical surgery (R0) including abdominoperineal resection (APR), LAR, or CAA; 2 (4.9%) received APR (R1) and then chemotherapy; 3 (7.3%) received LE or TEM; 2 (4.9%) received radiotherapy; 2 (4.9%) received palliative chemotherapy; and 3 (7.3%) for whom radical surgery was indicated refused it. Additionally, 3 of these patients were not able to undergo surgery because they had LR with concurrent DM. In the radical surgery group, 8.4% (29/344) experienced failure; 1.2% (4) had LR and 7.6% (26) had DM (Table 1). To calculate the LR and DM rates for each year of patient data, we summarized the data from patients with LR, DM, DFS, and OS according to specific time points (Tables 4 and 5).

**Table 4:** Local recurrence and distant metastasis after 1, 2, 3, and 5 years in observation and radical surgery groups

Studies (year)	No.of Patients		1-Year				2-Year				3-Year				4-Year				5-Year			
	OB	Surgery	LR (%)		DM (%)		LR (%)		DM (%)		LR (%)		DM (%)		LR (%)		DM (%)		LR (%)		DM (%)	
			OB	Surgery	OB	Surgery	OB	Surgery	OB	Surgery	OB	Surgery	OB	Surgery	OB	Surgery	OB	Surgery	OB	Surgery	OB	Surgery
Araujo et al [9](2015)	42	69	1(2.4)	0	2(4.8)	1(1.4)	4(9.5)	1(1.4)	3(7.0)	5(7.2)	8(19.0)	1(1.4)	6(14.3)	5(7.2)	8(19.0)	1(1.4)	7(16.7)	6	8(19.0)	1(1.4)	7(16.7)	7(10.1)
Li et al [2](2015)	30	92	0	0	0	0	1(3.3)	1(1.1)	0	0	2(6.7)	2(2.2)	0	1(1.1)	2(6.7)	2(2.2)	0	3(3.3)	2(6.7)	2(2.2)	1(3.3)	5(5.4)
Lee et al [13](2015)	8	28	2(2.5)	0	0	0	2(2.5)	1(3.6)	0	2(7.1)	2(2.5)	1(3.6)	0	3(10.7)	NS	NS	NS	NS	NS	NS	NS	NS
Smith RK et al [8] (2015)	18	30	1(5.6)	0	0	0	1(5.6)	0	0	1(3.3)	1(5.6)	0	1(5.6)	1(3.3)	1(5.6)	0	1(5.6)	1(3.3)	1(5.6)	0	1(5.6)	1(3.3)
Seshadri et al [11](2013)	23	10	5(21.7)	0	1(4.3)	NS	6(26.1)	0	1(4.3)	NS	7(30.4)	0	1(4.3)	NS	7(30.4)	0	2(8.7)	NS	7(30.4)	0	2(8.7)	NS
Smith JD et al [7](2012)	32	57	4(12.5)	0	1(3.1)	0	6(18.8)	0	2(6.2)	1(3.1)	—	—	—	—	—	—	—	—	—	—	—	—
Dalton et al [6](2012)	6	6	0	0	0	0	0	0	0	0	—	—	—	—	—	—	—	—	—	—	—	—
Maas et al [5](2011)	21	20	0	0	0	0	1(4.8)	0	0	0	—	—	—	—	—	—	—	—	—	—	—	—
Habr-Gama et al [4](2004)	71	22	0	0	0	0	0	0	1(1.4)	3(13.6)	0	0	1(1.4)	3(13.6)	0	0	2(2.8)	3(13.6)	1(1.4)	0	2(2.8)	3(13.6)

**Table 5:** Long-term survival in the observation and radical surgery groups of included studies

Studies (year)	NO.of Patients		1-Year Survival (%)				2-Year Survival (%)				3-Year Survival (%)				4-Year Survival (%)				5-Year Survival (%)			
			DFS		OS		DFS		OS		DFS		OS		DFS		OS		DFS		OS	
	OB	Surgery	OB	Surgery	OB	Surgery	OB	Surgery	OB	Surgery	OB	Surgery	OB	Surgery	OB	Surgery	OB	Surgery	OB	Surgery	OB	Surgery
Araujo et al [9](2015)	42	69	40(95.2)	68(98.6)	NS	NS	36(85.7)	62(89.9)	NS	NS	31(73.8)	63(91.3)	NS	NS	30(71.4)	62(89.9)	NS	NS	26(60.9)	57(82.8)	30(71.6)	62(89.9)
Li et al [2](2015)	30	92	30(100.0)	92(100.0)	30(100.0)	92(100.0)	29(96.7)	91(98.9)	30(100.0)	92(100.0)	28(93.3)	89(96.7)	30(100.0)	92(100.0)	28(93.3)	87(94.6)	30(100.0)	90(97.8)	27(90.0)	85(92.4)	30(100.0)	88(95.7)
Lee et al [13](2015)	8	28	6(75.0)	28(100.0)	NS	NS	6(75)	25(89.3)	NS	NS	6(75.0)	24(85.7)	NS	NS	NS	NS	NS	NS	NS	NS	NS	NS
Smith RK [8]et al (2015)	18	30	17(94.4)	0	18(100.0)	30(100.0)	17(94.4)	29(96.7)	18(100.0)	30(100.0)	16(88.9)	29(96.7)	18(100.0)	29(96.7)	16(88.9)	29(96.7)	18(100.0)	29(96.7)	16(88.9)	29(96.7)	18(100.0)	29(96.7)
Seshadri et al [11](2013)	23	10	17(73.9)	NS	23(100.0)	NS	16(69.6)	NS	21(91.3)	NS	15(65.2)	NS	20(87.0)	NS	14(60.9)	NS	17(73.9)	NS	14(60.9)	NS	17(73.9)	NS
Smith JD et al [7](2012)	32	57	28(87.5)	57(100.0)	32(100.0)	57(100.0)	28(87.5)	56(98.2)	31(96.9)	57(100.0)	—	—	—	—	—	—	—	—	—	—	—	—
Dalton et al [6](2012)	6	6	6(100.0)	6(100.0)	6(100.0)	6(100.0)	6(100.0)	6(100.0)	6(100.0)	6(100.0)	—	—	—	—	—	—	—	—	—	—	—	—
Maas et al [5](2011)	21	20	21(100.0)	19(98.0)	21(100.0)	19(98.0)	19(90.5)	19(98.0)	21(100.0)	19(98.0)	—	—	—	—	—	—	—	—	—	—	—	—
Habr-Gama et al [4](2004)	71	22	71(100.0)	22(100.0)	71(100.0)	22(100.0)	70(98.6)	19(86.4)	71(100.0)	20(90.9)	70(98.6)	19(86.4)	71(100.0)	20(90.9)	69(97.2)	19(86.4)	71(100.0)	20(90.9)	68(95.8)	19(86.4)	71(100.0)	20(90.9)

**Figure 3:**
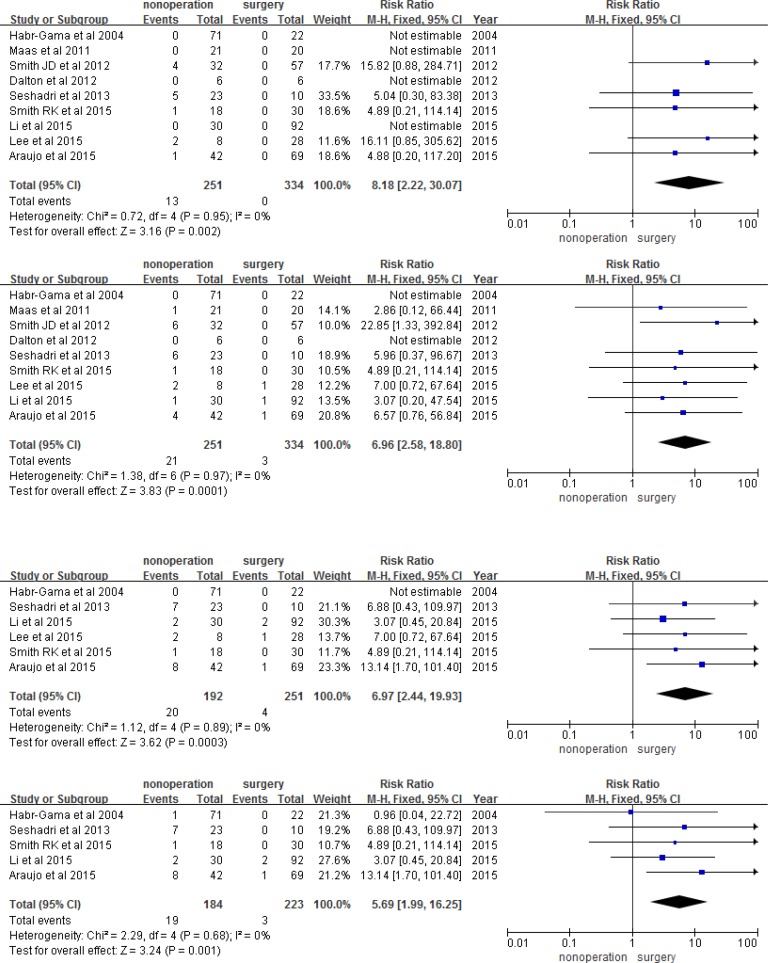
1, 2, 3, and 5-year local recurrence

**Figure 4:**
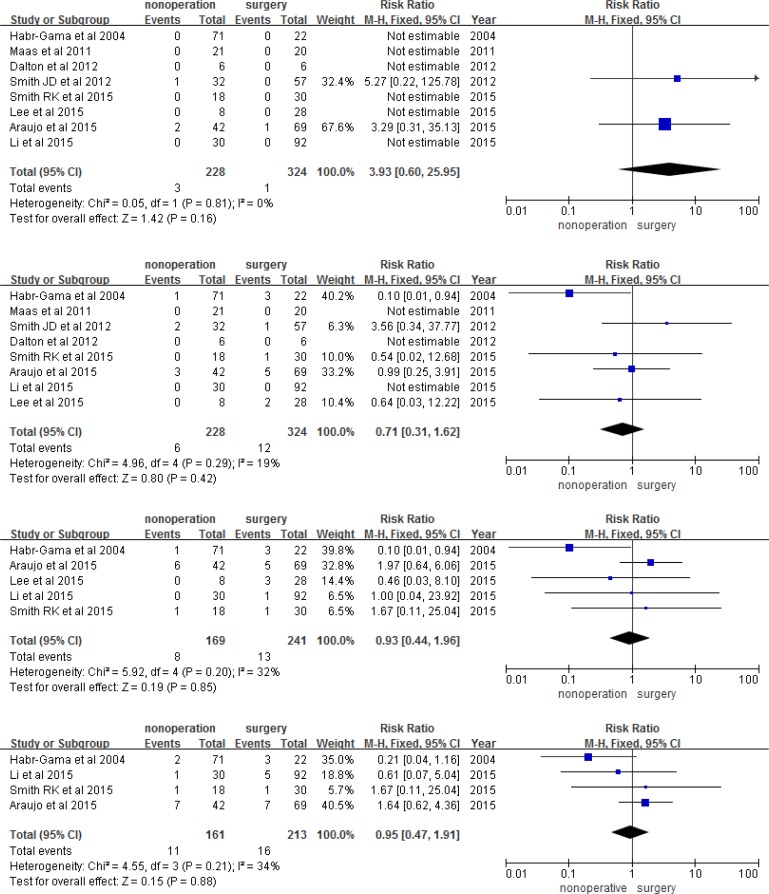
1, 2, 3, and 5-year distant metastasis

**Figure 5:**
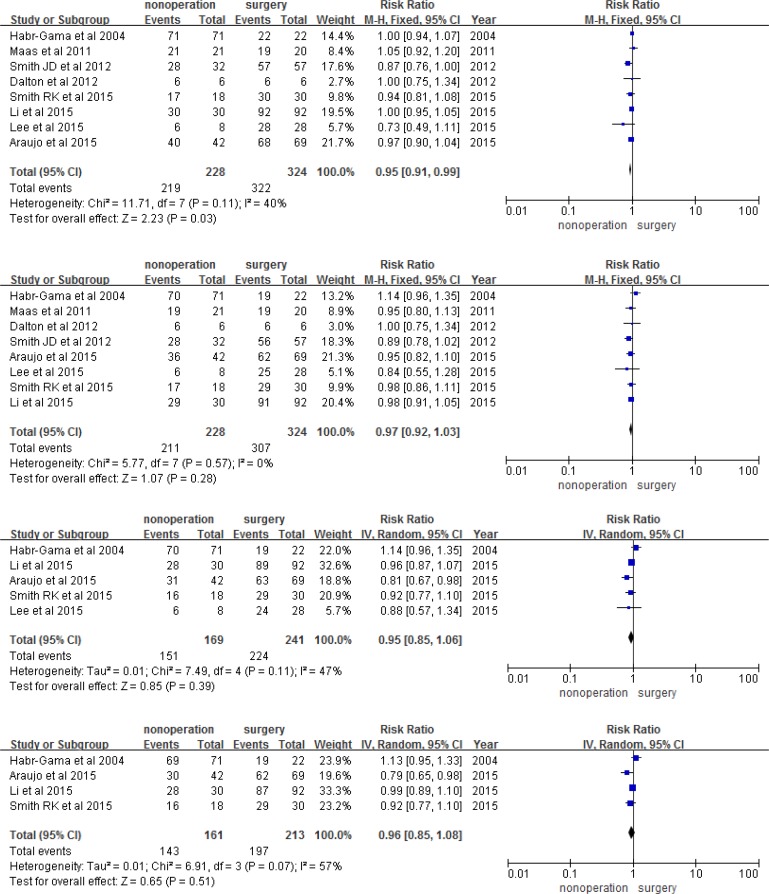
1, 2, 3, and 5-year disease free survival

**Figure 6:**
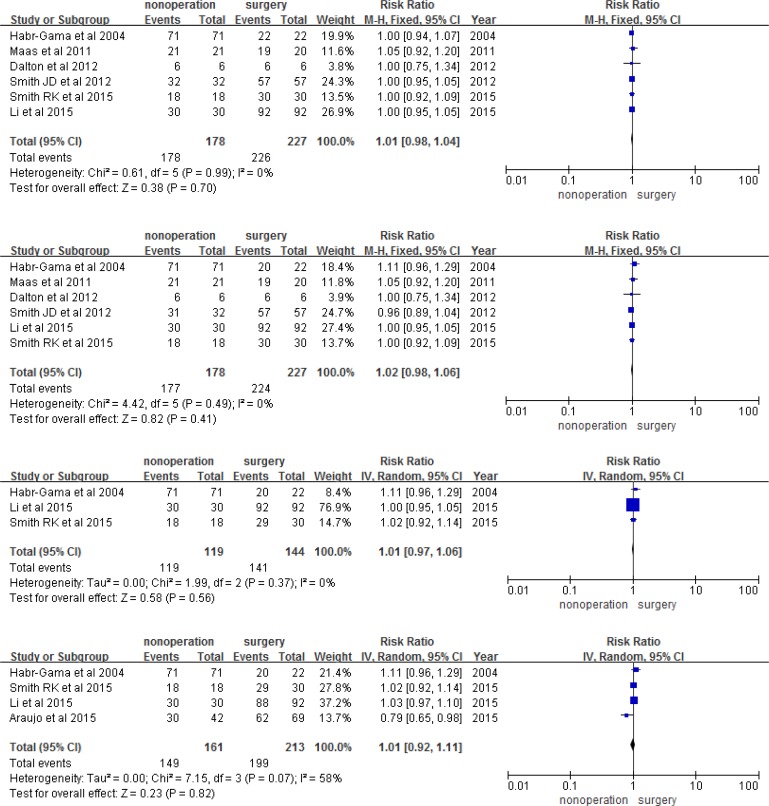
1, 2, 3, and 5-year overall survival

Using meta-analysis, we found that the observation group had a higher risk of 1, 2, 3, and 5-year LR than the surgery group (RR 8.18, 95% CI 2.22-30.07, *P* = 0.002; RR 6.96, 95% CI 2.58-56.84, *P* = 0.0001; RR 6.97, 95% CI 2.44-19.93, *P* = 0.003; RR 5.69, 95% CI 1.99-16.25, *P* = 0.001; respectively; Figure 3). However, the two groups had a similar risk of DM, DFS, and OS in each year (Figures 4, 5, and 6). The risk of 1, 2, 3, and 5-year DM was similar in nonoperation and radical surgery groups (RR 3.93, 95% CI 0.60-0.25.95, *P* = 0.160; RR 0.71, 95% CI 0.31-1.62, *P* = 0.420; RR 0.93, 95% CI 0.44-1.96, *P* = 0.12; RR 0.95, 95% CI 0.47-1.91, *P* = 0.88, respectively). Two articles that did not mention DFS and OS after surgery were excluded from this analysis [11, 13]. Patients in the observation and surgery groups had similar 1, 2, 3, and 5-year DFS (RR 0.95, 95% CI 0.91-0.99, *P* = 2.23; RR 0.97, 95% CI 0.92-1.03, *P* = 0.280; RR 0.95, 95% CI 0.85-1.06, *P* = 0.39; RR 0.96, 95% CI 0.85-1.08, *P* = 0.850, respectively). 3.6% (19/526) of patients died during the course of follow-up visits, mainly due to rectal tumor disease; 5.5% (12/220) of observation group patients died, while 2.3% (7/306) of surgery group patients died. The observation and surgery groups did not differ in 1, 2, 3, and 5-year OS (RR 1.01, 95% CI 0.98-1.04, *P* = 0.700; RR 0.1.02, 95% CI 0.98-1.06, *P* = 0.410; RR 0.95, 95% CI 0.97-1.06, *P* = 0.560; RR 1.01, 95% CI 0.92-1.10, *P* = 0.820, respectively).

## DISCUSSION

Previous studies indicate that pCR is predictive of good prognosis. In a pooled analysis of 484 patients with pCR, Mass [30] demonstrated that the 5-year DFS rates in patients with or without pCR were 83.3% and 65.6%, respectively (HR 0.44, 95% CI 0.34-0.57; *P* < 0.0001). For patients with pCR without residual tumor cells in the rectal wall and nodes, it has been debated widely whether radical surgery is necessary. Following NCRT, evidence from digital rectal examinations (DRE), MRIs, and endoscopies with biopsy and transrectal ultrasounds indicates that cCR is attained in about 26.8% of patients [31, 32]. Recently, however, Habr-Gama and colleagues [3] reported that 68.1% (47/69) of patients had cCR in that study.

Achieving cCR may allow patients to avoid radical surgery, which is accompanied by the risk of complications and mortality [33]. Additionally, although NCRT can help distal rectal cancer patients avoid excision of the anal sphincter, a notable proportion of patients required APR and permanent colostomies. However, even patients who undergo LAR to keep the anal sphincter have high rates of incontinence, anal mucus loss, anal blood loss, and daily pad use [34–36]. A modest but significant proportion of patients who have completed NCRT and have sustained cCR may be able to avoid radical surgery and associated complications if a wait-and-see policy is employed, although some of these patients may still require salvage radical surgery because of LR or DM [37]. A wait-and-see policy would also benefit patients with cCR who refuse surgery because of religious reasons, fistulas, or poor physical condition [2]. The wait-and-see policy for rectal cancer patients with a cCR after NCRT is based on careful selection and follow-up using endoscopy and up-to-date imaging, and appears both feasible and safe. The Brazil study [4] was the first to propose that nonoperation management could be used for patients with cCR. The Brazil study series [3, 4, 38, 39] also improved the process for nonoperation management, including patient selection, how and when to identify cCR, follow-up schedule, and salvage treatment. In the present study, we found that there is no difference in long-term survival, as measured by DM, DFS, and OS, in patients with cCR treated with a wait-and-see strategy compared to those with pCR who underwent radical surgery. Nonoperation management is, however, associated with a higher risk of LR. In the result of this meta-analysis the reason for patients treated by observation management had a LR rate but a similar DFS rate is that one study from Seshadri et al [11] with a higher risk of LR were excluded because of lack of the data of DFS. Although it is effective in many cases, the wait-and-see treatment strategy still needs to be improved.

Currently, a limitation of nonoperation management is the possibility of poor correlations between clinical findings and final pathologic findings from resected specimens. For example, cCR does not always correspond to pCR as indicated by DRE, CT, PET/CT, MRI, endoscopy with biopsy, and TU. Patients showing cCR who do not have pCR and who are not treated with radical surgery likely have a high risk of recurrence. Previous studies, including ours, clearly show that cCR does not always indicate pCR, and moreover, following NCRT, up to 7% of patients with pCR may have an incomplete clinical response characterized by residual rectal ulcers [2, 29].

Another obstacle for nonoperation management is that it does not address residual tumor cells that may remain in perirectal nodes, including lymph nodes and tumor deposits. Recently, the downstaging of nodes invaded has been examined by some authors. Perez [40] reported that histologic regression can be observed in nodes after NCRT. Moreover, the primary tumor regression grade (TRG) may predict lymph node response (LRG) [41–44]. Thus, the presence of residual tumor cells in nodes may be predicted by tumor response within the rectum. Indeed, most studies of observation management demonstrate an extremely low rate of recurrence in perirectal nodes [2–10]. However, CTs, MRIs, and transrectal ultrasounds are still crucial in determining node status when confirming cCR.

Until now, there is no standard guideline regarding of patients selection, when and how to perform nonoperation management for those with a cCR after NCRT. Firstly, Clinicians should select patients who may have a higher priority to perform the nonoperation management. Recent studies have established some guidelines for the selection of patients who are candidates for nonoperation management. First, the primary tumor should be located within 7 cm of the anal verge, which would be identifiable *via* DRE [38]. Second, Habr-Gama et al. [38] reported that only patients with tumor sizes of less than 7 cm should be considered for a wait-and-see policy. They recommended that these patients should be treated with radical surgery.

Secondly, early identification of cCR is also a key for ensuring the feasibility and safety of wait-and-see treatment. Initially, Habr-Gama et al. [26] achieved cCR using DRE, endoscopy, and excision of the residual scar; later, they focused on establishing more standardized requirements for cCR. In 2013, this team [3] proposed that the absence of residual ulceration, mass, or significant rectal wall irregularities as identified by MRI, PET/CT, or TU, in addition to CEA levels before and after NCRT, DRE, and endoscopy with biopsy (any residual scar, ulcer, or even local excision) be used to define cCR. Any ulcers, palpable nodules, or significant stenosis would suggest that cCR was not achieved. Habr-Gama et al. [31] suggested that patients with rectal cancer within 7 cm of the anal verge were suitable for cCR assessment; DRE accuracy in assessing this distance can reach 50% and is helpful for estimating cCR.

Thirdly, the time interval between NCRT and response assessment is critical. Most studies examined here assessed response between 6 and 10 weeks after treatment. Moore et al. [45] found that longer intervals were associated with much higher rates of complete tumor response in rectal cancer patients. A study of 1, 593 rectal patients from a Dutch hospital found that pCR rates were highest after a 10- to 11-week interval, and pCR rates did not increase at longer intervals [46]. A recent cohort study including 122 cases with cCR reported that in the surgery group, 5 of the 11 non-pCR patients had a TRG of 0 and were LN+, while 6 had a TRG of 1 without positive lymph nodes (minor residual tumor cells) [2]. Interestingly, the pCR and non-pCR patients had similar 5-year failure (LR and/or DM) rates (*P* = 0.350). This result might also be explained by an insufficient time interval (6-8 weeks) between NCRT completion and surgery, as further tumor cell necrosis and death might have occurred if the interval was longer [18]. Habr-Gama and colleagues have also suggested using an interval longer than 6 weeks for the assessment of residual disease in both primary tumors and perirectal nodes [20, 47, 48]. Thus, 8-11 weeks post-NCRT may be the optimal interval for identifying cCR.

Fourthly, the success of the wait-and-see strategy depends on a sustained cCR. Reports from Habr-Gama's group and others are not consistent regarding the time point for assessment of sustained cCR, which ranged from 12 to 14 months after NCRT completion, or regarding the time point for identifying patients failing to maintain cCR [2, 3, 5, 6, 8, 9, 20]. Only seven studies established a rigorous follow-up system (Table 6), and they suggested that cCR should only be considered sustained after at least 12 months. Although the timing is uncertain, a comprehensive and effective set of tools, including DRE, MRI, endoscopy plus biopsy (any residual scar tissue), PET/CT, TU, and CEA levels, is available for assessing cCR. Additionally, timely identification of failure to maintain cCR might render salvage treatments more effective. Until now, because the use of nonoperation management with short follow-up times has been relatively rare, it has not been clear that salvage treatments are safe and effective for patients with LR or/and DM. Surgical salvage might be the most effective way to cure patients with LR and resectable DM. In the studies by Habr-Gama et al.[14], most patients were treated by surgical salvage, including APR, LAR, and FTLE, but other studies reported that up to 25% could not be treated with salvage surgery [25, 28]. However, these later studies did not perform regular follow-ups, which may have delayed the detection of LR and DM, in turn reducing the viability of salvage surgery treatment. Thus, meticulous follow-up assessments may be crucial to the success of wait-and-see treatment strategies.

**Table 6:** Follow-up schedules for confirming initial and sustained cCR in included studies

Li et al, 2015	Mass et al, 2011	Dalton et al, 2012
**Initial (8-10 weeks)**	**Initial (6-8 weeks)**	**Initial (6-8 weeks)**
DRE, CEA, endoscopy, TU, CT (abdomen, and pelvis), MRI, chest X-ray	DRE, CEA, endoscopy, CT (abdomen, and pelvis), MRI	DRE, CEA, endoscopy, CT( abdomen, and pelvis), MRI or TU
**Year 1**	**Year 1**	**Year 1**
Every month: DRE, CEA	Every 3 months: DRE, CEA, endoscopy, MRI	At 3 months and 1 year: endoscopy(EUA), CEA
Every 3 months: endoscopy, TU	Every 6 months: CT	at 6 months: PET/CT, MRI
Every 6 months: CT, MRI, chest X-ray	**Year 2-3**	**Year ≥ 2**
**Year 2-3**	Every 3 months: CEA	Every 1year: PET/CT, MRI
Every 6 months: DRE, CEA, CT, MRI, endoscopy, chest X-ray, TU	Every 6 months: DRE, endoscopy, MRI	CEA levels were detected (duration: unclear)
**Year ≥ 4**	Every 1 year: CT	**Lee et al, 2015**
Every 1 year: DRE, CEA, CT, MRI, endoscopy, chest X-ray, TU	**Year 4-5**	**Initial (6 weeks)**
**Habr-Gama et al, 2013**	Every 6 months: DRE, CEA, endoscopy, MRI	MRI only
**Initial (10 weeks)**	Every 1 year: CT	**Year 1-2**
endoscopy (biopsy if possible; full-thickness excision, partial), MRI, PET/CT	**Smith et al, 2015**	Every 3 months: CEA, chest X-ray or CT, CT, MRI, endoscopy, PET
**Year 1**	**Initial (7-24 weeks)**	**Year3-5**
Every 2 month: DRE, CEA, endoscopy	DRE, endoscopy, MRI, TU, PET/CT or CT (thorax, abdomen and pelvis)	Every 6 months: CEA, chest X-ray or CT, CT, MRI, endoscopy, PET
Every 6 month: CT, PET/CT, chest X-ray	**Year 1**	**Araujo et al, 2015**
**Year 2**	Every 3 months: endoscopy, CEA	**Initial (duration not stated)**
Every 3-4 month: DRE, CEA, endoscopy	Every 1 year: endoscopy, CEA, PET/CT or CT	DRE, CEA, endoscopy, RMI
Every 6 month: CT, PET/CT, chest X-ray	**Year 2-3**	**Year 1-2**
**Year 3-5**	Every 6 months: endoscopy, CEA	Every 3 months: DRE, CEA, endoscopy, RMI(initial)
Every 6 month: DRE, CEA, endoscopy	**Year ≥ 4**	**year 3-5**
Every 1 year: CT, PET/CT, chest X-ray(only the third year)	Every 1 year: endoscopy, CEA, PET/CT or CT	Every 6 months: DRE, CEA, endoscopy

Abbreviations: DRE, digital rectal examination; CEA, carcinoembryonic antigen; CT, computed tomography; MRI, magnetic resonance; TU, transrectal ultrasound; PET/CT, positron-emission tomography/computed tomography.

Notes: The chest X-ray and chest CT is alternative. Biopsy is recommended if possible when endoscopy is performed in most of the studies. Li et al. and Habr-Gama et al. emphasize the importance of DRE in particular when confirming cCR.

Although our present study provides valuable information regarding the efficacy of nonoperation management in rectal cancer after NCRT, future studies should address some of its limitations. For example, meta-analysis of aggregate data does not allow for the examination of some factors that can be explored in meta-analysis of individual patient data, including differences among patient subgroups [49]. Additionally, there is a high risk of comparability bias in the 9 comparative studies we evaluated in the present meta-analysis. Furthermore, all of the studies examined used different wait-and-see treatment strategies. Finally, and perhaps most importantly, all of the studies examined here were nonrandomized and relatively small-scale. Regardless, our findings suggest that wait-and-see strategies should be evaluated in larger studies, which will help clarify the potential benefits of nonoperation management in rectal cancer patients.

## MATERIALS AND METHODS

We searched the electronic PubMed, Medline, and Embase databases for relevant articles and international meeting databases, including ECCO, ESTRO, and ESSO for abstracts published by October 1 2015. We searched for “rectal cancer” and “clinical complete response”, and all relevant keyword variations were used for both terms. Studies were included if: they were published in English; patients with local rectal cancer (cTNM stage: I to III) received radiotherapy with or without concurrent chemotherapy and achieved cCR; patients with cCR were treated with a wait-and-see strategy; data and time to event for LR, DM, DFS, and OS were provided. Studies without our primary end point, with previously irradiated patients, and case reports related to nonoperation management were excluded.

One reviewer (LJL) checked the titles and abstracts of the identified studies to select studies potentially meeting the inclusion criteria related to this topic. Two independent reviewers (SSX and YNY) examined full text copies of initially selected studies to decide which met the inclusion criteria. Two additional reviewers traced studies which were cited by the selected studies. Finally, two corresponding authors (JL and LJL) reviewed the selected studies to confirm their relevance.

### Outcomes

The primary endpoint of interest was local LR. Secondary endpoints were DM, DFS, and OS. All time-to-event variables were calculated from the date of NCRT completion. DFS was defined as time to any LR or/and DM. OS was defined as time from NCRT completion to death from any cause, or to end of follow-up (censored) according to included studies. All LR or/and DM events were defined as failures.

### Risk of bias assessment

We used the Newcastle-Ottawa Scale to measure the methodologic quality and risk of bias of the nonrandomized studies, including risk of bias in the selection and comparability of cohorts and outcomes [50]. The two independent reviewers (SSX and YNY) conducted the risk of bias assessment.

### Statistical analysis

We assessed heterogeneity using Cochran's Q statistic, and heterogeneity was considered statistically significant when *P* < 0.10 and the *I*^2^ > 50% [51]. We used the fixed-effect model with Mantel-Haenszel method to calculate summarized relative risk (RR) and 95 % CI. When significant heterogeneity existed, we used the random-effects method (Inverse Variance) to calculate summarized RR and 95% CI [52]. We assessed publication bias by funnel plots [53]. For all tests except for heterogeneity, a probability level < 0.05 was considered statistically significant. All calculations and graphs of LR, DM, DFS, and 1, 2, 3, and 5-year OS were completed using Review Manager 5.3 (The Nordic Cochrane Centre, The Cochrane Collaboration, 2014).
